# Decarbonization of the Italian healthcare system and European funds. A lost opportunity?

**DOI:** 10.3389/fpubh.2022.1037122

**Published:** 2022-12-14

**Authors:** Benedetta Armocida, Beatrice Formenti, Silvia Ussai, Eduardo Missoni, Chiara De Marchi, Massimiliano Panella, Graziano Onder, Laura Mancini, Marco Pistis, Marco Martuzzi, Francesco Barone-Adesi

**Affiliations:** ^1^Department of Cardiovascular, Endocrine-metabolic Diseases and Aging, Istituto Superiore di Sanità, Rome, Italy; ^2^Associazione di Promozione Sociale, Saluteglobale.it, Brescia, Italy; ^3^Clinical Pharmacology and Toxicology, University of Cagliari, Cagliari, Italy; ^4^CERGAS—Centre for Research on Health and Social Care Management, Bocconi University, Milan, Italy; ^5^Department of Public Health and Infectious Diseases, Sapienza University, Rome, Italy; ^6^Department of Translational Medicine, Università del Piemonte Orientale, Novara, Italy; ^7^Department of Environment and Health, Italian Institute of Health, Rome, Italy; ^8^Department of Biomedical Sciences, Section of Neuroscience and Clinical Pharmacology, University of Cagliari, Cagliari, Italy; ^9^CRIMEDIM-Center for Research and Training in Disaster Medicine, Humanitarian Aid and Global Health, Università del Piemonte Orientale, Novara, Italy

**Keywords:** climate change, healthcare carbon footprint, healthcare decarbonization, planetary health, public health policies

## Introduction

“Primum non nocere” is a basic tenet of medical practice, and the concepts of precaution and prevention have always been central in public health practice. However, mounting evidence shows that the health sector is a major contributor to climate change, which in turn is expected to cause a global health emergency in the next years (1). A recent report showed that the world healthcare systems' climate footprint equals 514 coal-fired power plants, accounting for 4.4% of global greenhouse gas (GHG) emissions. According to these estimates, if the global healthcare system were considered a single country, it would be the fifth largest emitter on the planet (1). This underlines the importance of including the evaluation of the environmental impact of healthcare systems in the global agenda. In this spirit, the European Academies' Science Advisory Council and Federation of European Academies of Medicine have recently called for an urgent need for the healthcare system itself to achieve ambitious targets of decarbonization across the EU and beyond (2).

## Global, European, and Italian policies

In the last UN Climate Change Conference in Glasgow (COP26), fifty countries committed to developing low-carbon health systems, and fourteen of them have also set a target date to reach net zero carbon emissions on or before 2050 (3). These countries follow the path set by the UK, which was the first to commit its NHS to becoming “carbon net zero,” adopting interventions such as increasing community care, greening the vehicle fleet, reducing waste of consumables, constructing new hospitals with net-zero emissions, and training health staff on energy conservation (4).

The EU addresses the issue of climate change through various policies, mainly within the framework of the European Green Deal (5). However, in the case of healthcare systems, the emphasis is largely placed on adaptation to climate change (e.g., Article 5 of the European Climate Law) rather than on the mitigation of emissions (5). This could partly explain why only six EU countries (Belgium, Germany, Ireland, the Netherlands, Norway, and Spain) have submitted formal commitments to the COP26 Presidency to strengthen and develop sustainable healthcare systems (3). In these terms, EU countries could instead take advantage of already existing schemes such as (i) the Pharmaceutical Strategy for Europe, which underlines the importance of using environmentally sustainable and climate-neutral pharmaceuticals, (ii) the eHealth, the digital health strategy, which although specifically outlines improvements in access to care and its quality, could also have an important role in the decarbonization of healthcare systems, and (iii) the Farm to Fork Strategy, which can support the use of healthy and sustainable food in the healthcare system (2).

Notably, Italy is not on the list of signatory countries even though (i) its healthcare footprint is 4% of the national footprint (1); (ii) the country is currently investing in ecological transition; (iii) and climate policies promote infrastructure improvements at the national level and energy efficiency improvements at the regional level in public sector buildings, including hospitals. Moreover, despite the Italian National Prevention Plan recognizing that the health sector must contribute to climate change mitigation and improve its resilience (6), this sector is not explicitly mentioned when planned emission reductions are indicated.

The opportunity has also been missed in the implementation of the National Recovery and Resilience Plan (NRRP), which is supported by the EU with €68.9 billion in grants and €122.6 billion in loans, among which about 38% should support climate objectives (7). Indeed, although the NRRP aims for a progressive decarbonization of “all sectors” in Italy, the healthcare system is never explicitly mentioned. This is particularly striking, considering that two of the assets of NRRP are the strengthening of primary healthcare and the development of telemedicine, both of which, replacing hospital-based services, can become instrumental to reduce emissions as well (7). It is also noteworthy that in Italy, 82% of constructions were built before 1990, and 58% before 1970, well before the enactment of the law L10/1991, through which rational energy use began to be significantly regulated (8). Given these data, the road to greater community and home-health, in line with the NRRP, seems decisive to improve energy efficiency and, together with telemedicine, to decrease the demand on the supply chain by reducing and/or replacing hospital-based services (7). In this context, the investment foreseen in the NRRP for the development of community-oriented technical, and digital skills for healthcare professionals could also be strategic (7).

## Discussion

As the healthcare system's contribution to the climate crisis is projected to further grow in the next years (1), specific decarbonization policies in this sector should be initiated and prioritized as soon as possible, using the existing schemes at the national and European levels. These should target the healthcare supply chain and its direct and indirect emissions, professional education, governance, and financing. Specifically, energy efficient infrastructure, toward power health care with 100% clean, renewable electricity and mitigation of temperature extremes in winters and summer; energy-saving and optimization (e.g., LED bulbs); green policies aimed to reduce the transportation emissions; encouragement of resource stewardship, food waste prevention and adoption of healthy, seasonal and sustainable diets; reduction of inefficient and unnecessary practices as well as unnecessary pharmaceutical use, while incentivizing the production of affordable climate-smart medicine and sustainable healthcare waste management (9).

Additionally, it would be essential to ensure the implementation of the monitoring system envisaged in the Ministerial Decree 77 (10), to direct specific interventions and decision-making, and to define the sustainability of NRRP, with an approach based on the integrated prevention, closely linked with community-based health care. Finally, dedicated research investments on the environmental impacts of the healthcare system and the costs and benefits of decarbonization interventions are warranted. Indeed, these are pivotal to direct policy-makers to effectively foster environmental sustainability and systems *for* health (11). These priorities should be framed around the principle of linking health to all-policies to (i) promote the circular economy and the bioeconomy, (ii) reduce GHG emissions also invest in the energy efficiency of existing facilities, and (iii) provide a more sustainable economic model.

## Author contributions

BA and FB-A conceptualized the Opinion. BA drafted the manuscript. BF, SU, EM, CD, MPa, GO, MPi, MM, LM, and FB-A contributed to reviewing and finalizing the opinion. All authors approved the final version of the manuscript.
